# Exposure to Doxorubicin Modulates the Cardiac Response to Isoproterenol in Male and Female Mice

**DOI:** 10.3390/ph16030391

**Published:** 2023-03-04

**Authors:** Kevin Agostinucci, Marianne K. O. Grant, Wongel Melaku, Chandini Nair, Beshay N. Zordoky

**Affiliations:** Department of Experimental and Clinical Pharmacology, College of Pharmacy, University of Minnesota, Minneapolis, MN 55455, USA

**Keywords:** doxorubicin, sex differences, isoproterenol, hypertrophy

## Abstract

Sex is a salient risk factor in the development of doxorubicin-induced cardiotoxicity. Sex differences in the heart’s ability to respond to hypertrophic stimuli in doxorubicin-exposed animals have not been reported. We identified the sexual dimorphic effects of isoproterenol in mice pre-exposed to doxorubicin. Male and female intact or gonadectomized C57BL/6N mice underwent five weekly intraperitoneal injections of 4 mg/kg doxorubicin followed by a five-week recovery period. Fourteen days of subcutaneous isoproterenol injections (10 mg/kg/day) were administered after the recovery period. Echocardiography was used to assess heart function one and five weeks after the last doxorubicin injection and on the fourteenth day of isoproterenol treatment. Thereafter, mice were euthanized, and the hearts were weighed and processed for histopathology and gene expression analysis. Doxorubicin did not produce overt cardiac dysfunction in male or female mice before starting isoproterenol treatment. The chronotropic response to a single isoproterenol injection was blunted by doxorubicin, but the inotropic response was maintained in both males and females. Pre-exposure to doxorubicin caused cardiac atrophy in both control and isoproterenol-treated male mice but not in female mice. Counterintuitively, pre-exposure to doxorubicin abrogated isoproterenol-induced cardiac fibrosis. However, there were no sex differences in the expression of markers of pathological hypertrophy, fibrosis, or inflammation. Gonadectomy did not reverse the sexually dimorphic effects of doxorubicin. Additionally, pre-exposure to doxorubicin abrogated the hypertrophic response to isoproterenol in castrated male mice but not in ovariectomized female mice. Therefore, pre-exposure to doxorubicin caused male-specific cardiac atrophy that persisted after isoproterenol treatment, which could not be prevented by gonadectomy.

## 1. Introduction

Doxorubicin (DOX) is an anthracycline chemotherapeutic agent that is used for the treatment of solid tumors and blood cancers [1]. The use of DOX in a cancer treatment plan has contributed to the reduction in cancer burden and the increased survivorship in cancer patients [2,3]. Although DOX has been shown to be an effective chemotherapeutic, its clinical utility is limited due to dose-limiting and irreversible cardiotoxicity [4]. Thus, understanding the mechanism of DOX-induced cardiotoxicity is important to improve the clinical utility of DOX for the treatment of cancer. Low, repeated doses of DOX have been shown to produce latent cardiotoxicity. Weekly DOX administration accumulating to 20 mg/kg in experimental animal models does not cause observable changes in cardiac function; however, it is associated with reductions in heart weight and increased markers of cardiotoxicity [5]. The latent cardiotoxic effect of DOX can predispose the heart to overt cardiomyopathy following a second cardiovascular insult [6]. Our prior work has shown that angiotensin II-induced hypertension worsens cardiovascular function in DOX-exposed mice [7,8]. Therefore, we are interested in identifying the mechanisms in which different cardiovascular pathologies could exacerbate DOX-induced cardiotoxicity in a two-hit manner. In our most recent work, we have shown that angiotensin II and isoproterenol (ISO) display divergent cardiac effects in adult mice that were treated with DOX (4 mg/kg/week for 3 weeks, a cumulative dose of 12 mg/kg) at a young age. Angiotensin II, a hypertensive pathologic stimulus, contributed to worsening cardiac function, fibrosis, and inflammation in DOX-exposed mice, while ISO did not exacerbate the latent DOX-induced cardiotoxicity [9].

Isoproterenol is a β-adrenergic agonist that induces cardiac stress without elevating blood pressure. Interventions that increase sympathetic drive such as dobutamine and exercise stress echocardiography have been used to detect latent cardiotoxicity in cancer survivors years after the completion of DOX treatment [10,11,12,13,14]. Since ISO displays similar cardiac actions to dobutamine and exercise, it was counterintuitive that ISO was not able to significantly exacerbate the latent cardiotoxic effects of DOX in our recent study that used the relatively low cumulative dose of 12 mg/kg. Therefore, in the current study, we sought to increase the cumulative dose of DOX to 20 mg/kg by administering five weekly doses of DOX 4 mg/kg to determine if ISO could uncover latent cardiotoxicity induced by this DOX dosage regimen. Noteworthy, our pilot studies demonstrate that DOX 4 mg/kg/week for 6 weeks, a cumulative dose of 24 mg/kg, caused cardiac dysfunction per se, indicating this dose is not suitable for our proposed two-hit studies.

Sex differences in DOX-mediated cardiotoxicity have been shown in pre-clinical and clinical models. Girls before puberty and post-menopausal women are at an increased risk of DOX-mediated cardiotoxicity compared to boys [15]. However, pre-menopausal women are less likely to exhibit severe cardiotoxicity after DOX exposure compared to men [16]. This observation suggests that sex hormones may be involved in the changes in the risk of DOX-mediated cardiotoxicity. We and others have demonstrated that female rodents are protected from both acute and chronic DOX-induced cardiotoxicity [17,18,19,20]. Nevertheless, the influence of sex as a risk factor for developing overt cardiotoxicity after undergoing a second cardiovascular hit in DOX-exposed animals has not been reported. Therefore, another objective of this study is to identify sex differences in the cardiac response of ISO in mice pre-exposed to DOX.

## 2. Results

### 2.1. Baseline Cardiac Function

**Figure 1:**
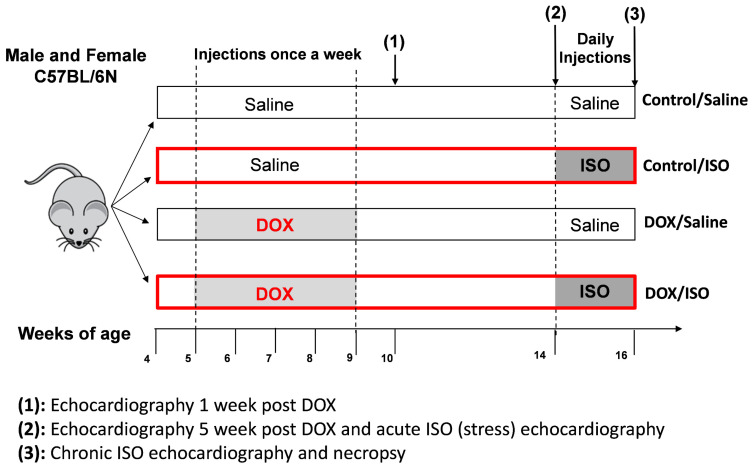
**Summary of the experimental design for DOX (4 mg/kg/week) and ISO (10 mg/kg/week) treatments.** Male and female intact or gonadectomized mice were administered DOX or an equal volume of saline (control) at five weeks of age once a week for five consecutive weeks using intraperitoneal injections. After the last dose of DOX, mice were not administered treatment for the following five weeks. One (1) and five (2) weeks after the last DOX injection, baseline heart function was assessed with echocardiography. The ISO challenge began after the five-week recovery period. ISO or an equal volume of saline was administered subcutaneously for 14 consecutive days. Stress (acute ISO) echocardiography (2) was measured five minutes after the first ISO injection. Terminal echocardiography (3) was measured one day after the last ISO injection. After the final assessment of heart function, all mice were sacrificed, and tissues were collected for biochemical analysis. Abbreviations: DOX, doxorubicin; ISO, isoproterenol.

Baseline cardiac function and morphometry were assessed one and five weeks after administration of the last DOX injection to determine the immediate and delayed effects of our DOX treatment regime before challenging the mice with catecholamine stress. The treatment protocol described in Figure 1 allows for repeated low-dose administration of DOX not exceeding the threshold of 24 mg/kg that causes overt cardiac dysfunction based on our pilot experiments and in agreement with published studies (5). Five weeks of DOX administration at a dose of 4 mg/kg/week did not cause significant changes in cardiac function one or five weeks after the last DOX treatment (Table 1). However, an assessment of cardiac morphometry shows significant reductions in left ventricle (LV) mass one week after the last DOX treatment in male mice only (Table 1). Furthermore, the diastolic LV anterior wall was significantly thinner in DOX-exposed males compared to saline-treated mice five weeks post-treatment (Table 1). In female mice, there were no significant differences in the parameters of cardiac function or morphometry (Table 1). Therefore, the DOX treatment protocol used in this experiment did not produce overt changes in cardiac function but left male mice with smaller LVs.

### 2.2. Acute ISO (Stress) Echocardiography

**Figure 2:**
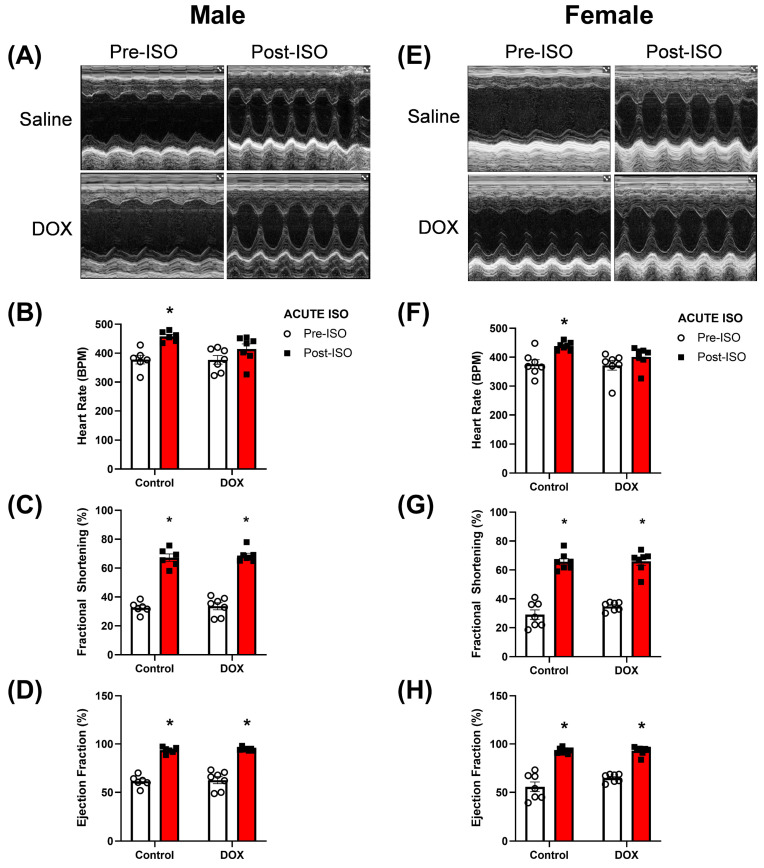
**Stress echocardiography did not show sex-related differences in DOX-exposed intact mice. Echocardiography was performed before and five minutes after the first ISO injection.** (**A**,**E**) Representative M-mode images, (**B**,**F**) heart rate, (**C**,**G**) fractional shortening, and (**D**,**H**) ejection fraction were assessed in intact males (n = 6–7) and females (n = 7), respectively. Values are represented as means ± SEM. Statistical significance of pairwise comparisons was determined using two-way ANOVA with Šidák’s post hoc analysis. Statistical significance was noted when *p* < 0.05 for comparisons between pre-acute ISO and post-acute ISO (*). Abbreviations: DOX, doxorubicin; ISO, isoproterenol.

Stress echocardiography was performed to reveal latent cardiotoxicity in mice pre-exposed to DOX. We used an experimental scheme where we administered 5 weekly injections of DOX 4 mg/kg followed by a 5-week recovery period before starting the stress echocardiography (Figure 1). Five minutes after one injection of ISO 10 mg/kg, male and female mice exhibited positive chronotropic and inotropic responses in the control group (Figure 2A–H). DOX-exposed male and female mice exhibited a blunted chronotropic response to acute ISO administration (Figure 2B,F). The positive inotropic response, evidenced by the increases in the fractional shortening and ejection fraction, was maintained after ISO administration in both male and female DOX-exposed mice (Figure 2C,D,G,H). There was no sex differences in the response to acute ISO administration in mice previously exposed to DOX. For each measured cardiac parameter, we took the difference between the post-ISO and pre-ISO values to determine the magnitude of change. When comparing the magnitude of ISO-induced changes, no differences were detected in heart rate, fractional shortening, and ejection fraction (Supplementary Figure S1).

### 2.3. Response to Chronic ISO Administration in Intact Mice

**Figure 3:**
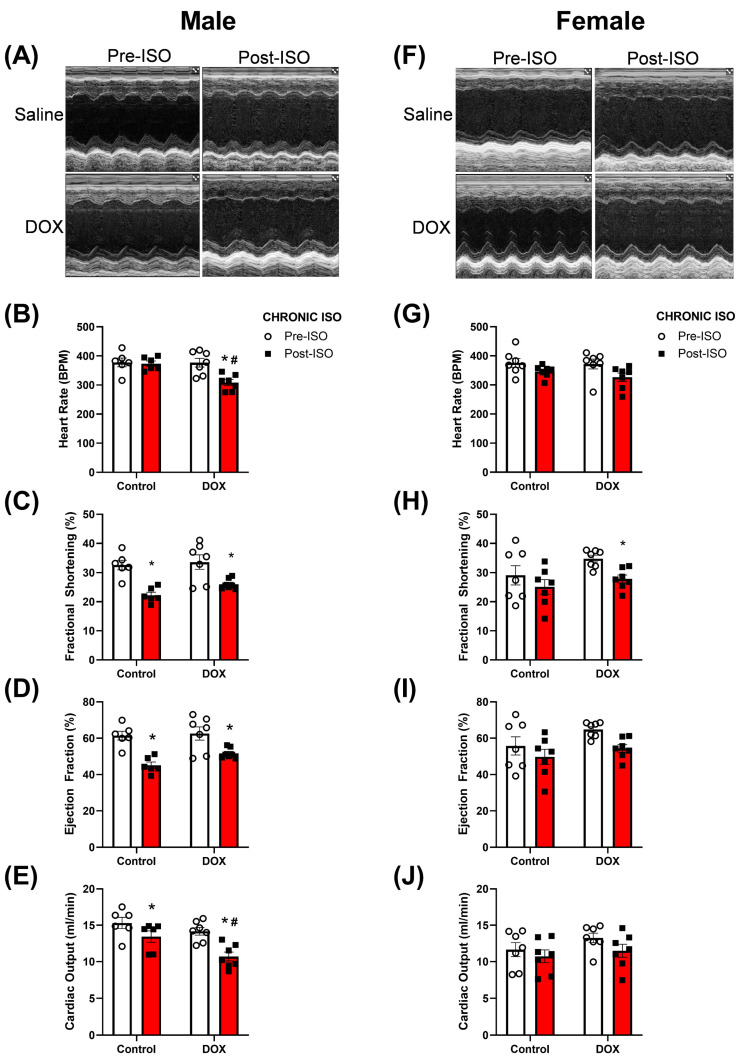
**Echocardiography revealed sex-related differences in response to chronic administration of ISO in DOX-exposed intact mice.** Echocardiography was performed before and after the 14-day ISO challenge. (**A**,**F**) Representative M-mode images, (**B**,**G**) heart rate, (**C**,**H**) fractional shortening, (**D**,**I**) ejection fraction, and (**E**,**J**) cardiac output were assessed in intact males (n = 6–7) and females (n = 7), respectively. Values are represented as means ± SEM. Statistical significance of pairwise comparisons was determined using two-way ANOVA with Šidák’s post hoc analysis. Statistical significance was noted when *p* < 0.05 for comparisons between pre-chronic ISO and post-chronic ISO (*) and DOX vs control (#). Abbreviations: DOX, doxorubicin; ISO, isoproterenol.

The same treatment protocol was used to determine if chronic administration of ISO for 14 days will exacerbate the cardiotoxicity induced by DOX (Figure 1). In male mice, the 14-day ISO challenge reduced fractional shortening and ejection fraction while not showing changes in heart rate (Figure 3A–D). DOX-exposed male mice exhibited a significant decline in heart rate after 14 days of ISO treatment (Figure 3B). The reduction in fractional shortening and ejection fraction in response to ISO in DOX-exposed mice appears to be similar to the control group (Figure 3C,D). Female mice, on the other hand, did not exhibit significant changes in heart rate, fractional shortening, or ejection fraction following 14 days of ISO administration (Figure 3F–I). Furthermore, heart rate and ejection fraction were not significantly different in DOX-exposed females, although fractional shortening was significantly reduced (Figure 3G–I). DOX-exposed males exhibited a more pronounced reduction in cardiac output after 14 days of ISO treatment (Figure 3E) while in female mice, there were no significant differences in cardiac output (Figure 3J). Again, when assessing the magnitude of ISO-induced changes in cardiac function, DOX-exposed male mice exhibited the largest change in heart rate compared to saline-treated males (Supplementary Figure S2A). For all other parameters, no significant differences were detected between male and female mice (Supplementary Figure S2B–D).

**Figure 4:**
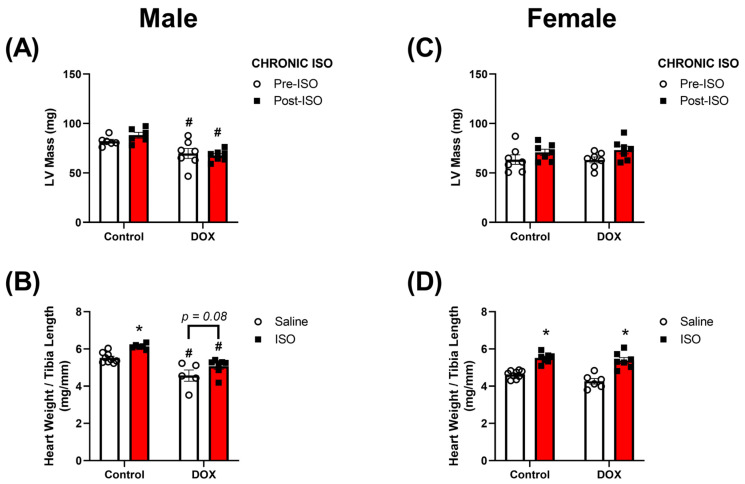
**Heart weights after 14 days of ISO challenge in male or female intact mice pre-exposed to DOX.** LV mass was assessed with echocardiography prior to and after the last ISO injection in (**A**) male (n = 6–7) and (**C**) female (n = 7) mice. Heart weight-to-tibia length was assessed at the end of the ISO challenge in (**B**) males (n = 5–9) and (**D**) females (n = 6–9). Values are represented as means ± SEM. Statistical significance of pairwise comparisons was determined using two-way ANOVA with Šidák’s post hoc analysis. Statistical significance was noted when *p* < 0.05 for comparisons between ISO and saline (*) and DOX vs control (#). Abbreviations: DOX, doxorubicin; ISO, isoproterenol; LV, left ventricle.

The LV mass was also measured to determine if DOX had an effect on the hypertrophic response of ISO. DOX-exposed male mice had significantly lower LV mass than control male mice even after ISO treatment (Figure 4A). There was no statistically significant difference in the magnitude of change in LV mass after ISO between saline and DOX groups (Supplementary Figure S2E). However, ISO treatment caused an increase in LV mass in all groups of mice except the DOX-exposed males (Supplementary Figure S2E). Similar to LV mass, heart weight/tibial length (HW/TL) was significantly lower in DOX-exposed male mice than that in the control (Figure 4B). ISO treatment caused a significant increase in HW/TL in control mice (Figure 4B). Although ISO treatment caused a modest increase in HW/TL in DOX-exposed mice, this effect was not statistically significant (*p* = 0.08) (Figure 4B). DOX-exposed females, on the other hand, were protected from DOX-induced reductions in LV mass (Figure 4C) and HW/TL (Figure 4D) and demonstrated a robust statistically significant hypertrophic response to ISO treatment similar to control female mice (Figure 4D).

**Figure 5:**
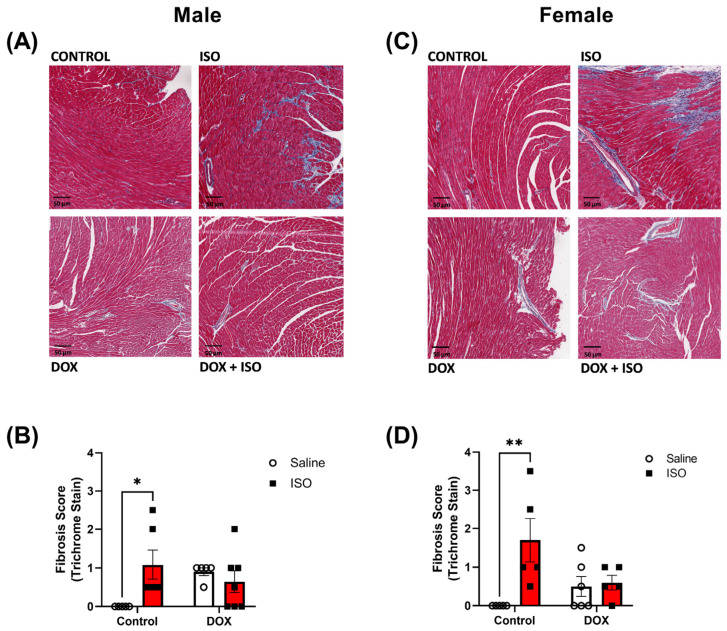
**Histopathologic evaluation of myocardial fibrosis after 14 days of ISO challenge in the hearts of intact male and female mice pre-exposed to DOX.** Representative images from Masson’s trichrome-stained heart sections in (**A**) male and (**C**) female mice; bar scale = 50 µM. Semi-quantification of fibrosis score derived from Masson’s trichrome stain in (**B**) male (n = 5–7) and (**D**) female (n = 5–6) hearts. Statistical significance of pairwise comparisons was determined using two-way ANOVA with Šidák’s post hoc analysis. (* *p* < 0.05, ** *p* < 0.01).

**Figure 6:**
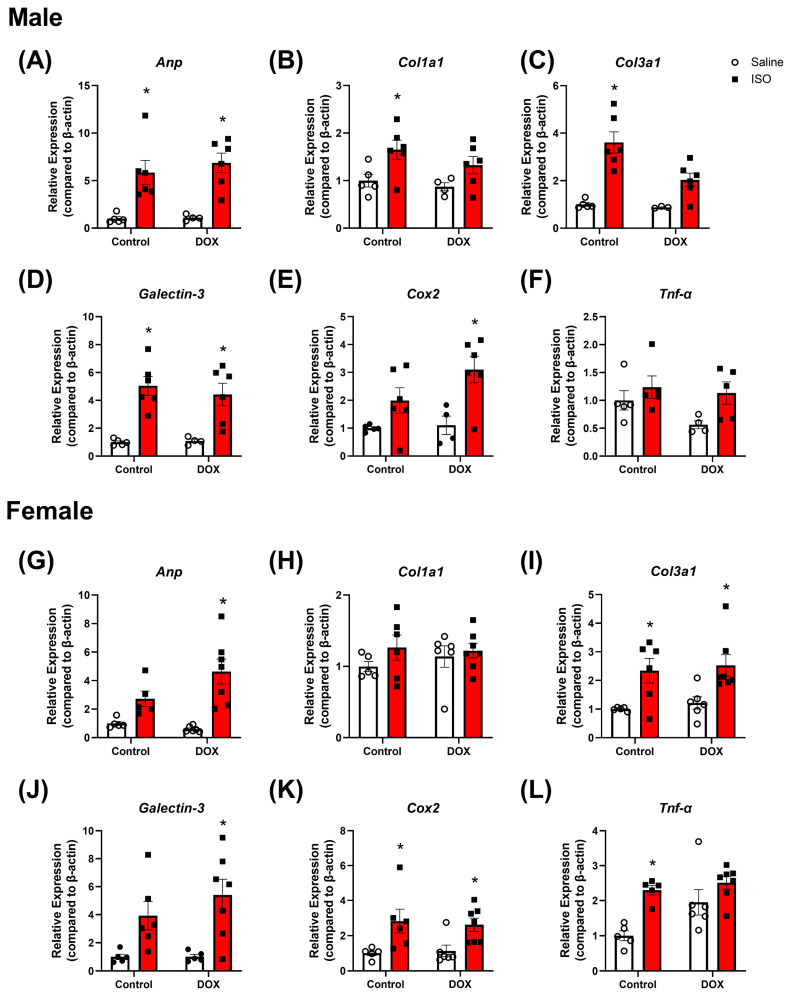
**Gene expression in the hearts of intact male and female mice pre-exposed to DOX after 14 days of ISO challenge.** The mRNA expression of (**A**,**G**) *Anp*, (**B**,**H**) *Col1a1*, (**C**,**I**) *Col3a1*, (**D**,**J**) *Galectin*-*3*, (**E**,**K**) *Cox2*, and (**F**,**L**) *TNF*-α was determined with real-time PCR (n = 4–7 per group); results were normalized to *beta-actin* and are expressed relative to the control group. Values are represented as means ± SEM. Statistical significance of pairwise comparisons was determined using two-way ANOVA with Šidák’s post hoc analysis. Statistical significance was noted when *p* < 0.05 for comparisons between ISO and saline (*). Abbreviations: DOX, doxorubicin; ISO, isoproterenol, Anp, atrial natriuretic peptide; Col1a1, collagen 1a1; Col3a1, collagen 3a1; Cox2, cyclooxygenase 2; TNF-α, tumor necrosis factor alpha.

To determine if the observed changes in cardiac function after the DOX and ISO treatment protocol are associated with pathological changes in the heart, histopathology and markers of cardiac damage, inflammation, and fibrosis were assessed. Analysis of Masson’s trichrome-stained heart sections revealed increased fibrosis in male and female mice following ISO treatment (Figure 5). DOX alone was not sufficient to cause a statistically significant effect on cardiac fibrosis. However, pre-exposure to DOX prevented the ISO-induced fibrosis observed in both male and female hearts (Figure 5B,D). Markers for pathologic cardiac hypertrophy (*Anp*), fibrosis (*Col1a1*, *Col3a1*, and *Galectin*-*3*), and inflammation (*Cox2* and *Tnf*-α) were also assessed (Figure 6A–L). Significant increases in *Anp* gene expression were observed in male and female mice pre-exposed to DOX following ISO treatment, suggesting cardiac pathology (Figure 6A,G). *Anp* was significantly increased by ISO alone in male but not female mice, and DOX alone did not significantly alter *Anp* expression in male or female mice (Figure 6A,G). Next, *Col1a1*, *Col3a1*, and *Galectin*-*3* gene expression were measured to determine changes in fibrotic markers in the heart. In males, ISO alone was capable of increasing the expression of all three markers of fibrosis (Figure 6B–D). However, in male mice pre-exposed to DOX, ISO was only able to significantly increase the expression of *Galectin*-*3* (Figure 6D). Female mice did not show the same trends in gene expression as males (Figure 6). *Col1a1* expression in female mice was not affected by ISO in the control or DOX groups (Figure 6H). *Col3a1* expression was significantly increased by ISO in both the control and DOX-treated female groups (Figure 6I). The expression of *Galectin*-*3* was increased by ISO in the control group (not significant) and significantly increased in DOX-treated female mice (Figure 6J). Lastly, *Cox2* and *Tnf*-α were used as markers of inflammation. Both markers were increased by ISO alone in female but not male mice (Figure 6E–F,K–L). In both male and female mice, DOX exposure prior to ISO administration resulted in a significant increase in *Cox2* (Figure 6E,K) but not *Tnf*-α (Figure 6F,L).

Collectively, DOX treatment caused cardiac atrophy in intact male mice, and partially diminished the hypertrophic effect of ISO. In contrast, intact female mice were protected from DOX-induced cardiac atrophy and demonstrated a robust hypertrophic response to ISO treatment. Counterintuitively, DOX treatment partially abrogated ISO-induced myocardial fibrosis in both males and females; however, it did not result in significant sex-specific changes in markers of pathologic hypertrophy, fibrosis, or inflammation.

### 2.4. Response to Chronic ISO Administration in Gonadectomized Mice

**Figure 7:**
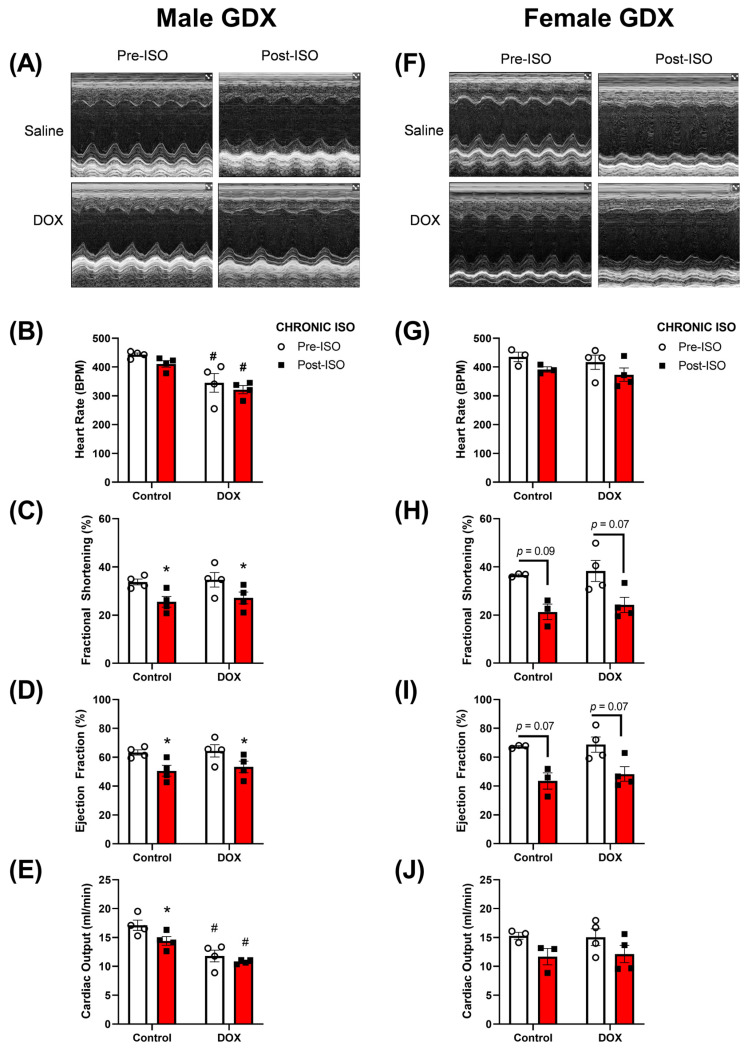
**Echocardiography revealed sex-related differences in DOX-exposed gonadectomized mice.** Cardiac function was assessed with echocardiography prior to and after the last ISO injection. (**A**,**F**) Representative M-mode images, (B,G) heart rate, (**C**,**H**) fractional shortening, (**D**,**I**) ejection fraction, and (**E**,**J**) cardiac output were assessed in gonadectomized males (n = 4) and females (n = 3–4) respectively. Values are represented as means ± SEM. Statistical significance of pairwise comparisons was determined using two-way ANOVA with Šidák’s post hoc analysis. Statistical significance was noted when *p* < 0.05 for comparisons between pre-chronic ISO and post-chronic ISO (*) and DOX vs control (#). Abbreviations: GDX, gonadectomized; DOX, doxorubicin; ISO, isoproterenol.

**Figure 8:**
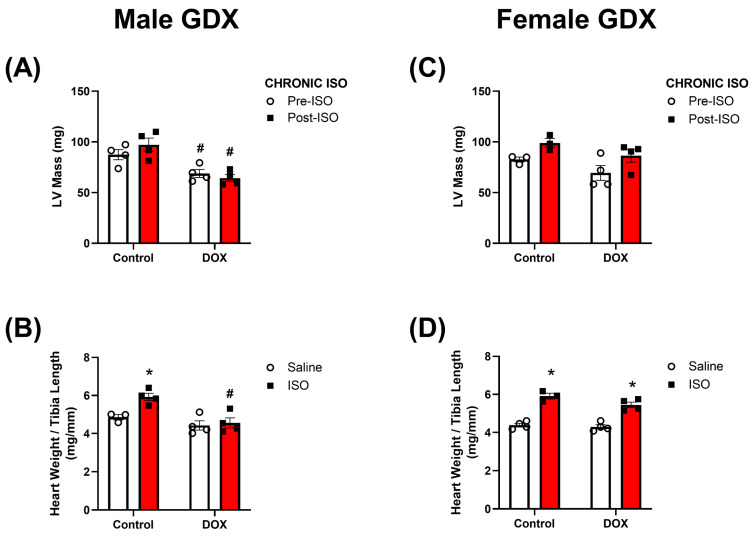
**Heart weights of gonadectomized mice after 14 days of ISO challenge.** LV mass was assessed with echocardiography prior to and after the last ISO injection in (*A*) male (n = 4) and (**C**) female (n = 3–4) gonadectomized mice. Heart weight-to-tibia length was assessed at the end of the ISO challenge in (*B*) males (n = 3–4) and (*D*) females (n = 3–4). Values are represented as means ± SEM. Statistical significance of pairwise comparisons was determined using two-way ANOVA with Šidák’s post hoc analysis. Statistical significance was noted when *p* < 0.05 for comparisons between ISO and saline (*) and DOX vs control (#). Abbreviations: GDX, gonadectomized; DOX, doxorubicin; ISO, isoproterenol; LV, left ventricle.

The experiments up to this point characterized the cardiotoxic effects of DOX in intact males and females after acute or chronic ISO treatment. To determine the relevance of sex hormones in mediating the observed sex differences, 5-week-old gonadectomized (GDX) male and female mice were administered DOX 4 mg/kg/week for 5 weeks, followed by a 5-week recovery period and then challenged with ISO 10 mg/kg/day for 14 days (Figure 1). The same parameters in cardiac function were assessed in these animals and the trends observed were generally similar to those observed in intact mice (Figure 3 and Figure 7). Furthermore, male GDX mice exhibited significant reductions in fractional shortening and ejection fraction after 14 days of ISO treatment in both the control and DOX groups (Figure 7C,D). GDX females exhibited a decline in fractional shortening and ejection fraction in response to chronic ISO treatment in the control and DOX groups, although this was not statistically significant (*p* < 0.1) (Figure 7H,I). DOX significantly reduced the heart rate and cardiac output in GDX male mice when compared to controls (Figure 7B,E), an effect that was not observed in intact male mice (Figure 3B,E). Fourteen days of ISO treatment did not change the heart rate in control GDX male mice and did not further reduce the heart rate in the DOX-exposed group (Figure 7B). In GDX female mice, no significant differences in heart rate or cardiac output were observed following DOX or ISO treatments (Figure 7G,J). There were no significant sex or DOX-mediated differences in the magnitude of ISO-induced changes in the measured cardiovascular parameters (Supplementary Figure S3). The changes in heart weight following the 14-day ISO challenge in GDX mice were similar to those observed in intact mice (Figure 4 and Figure 8). Fourteen days of ISO produced a hypertrophic response in control GDX male mice; however, pre-exposure to DOX completely abrogated the hypertrophic response to chronic ISO treatment in GDX male mice (Figure 8A,B). Similar to intact female mice, GDX female mice were protected from DOX-induced cardiac atrophy and exhibited a robust hypertrophic response to 14 days of ISO treatment in the control and DOX groups (Figure 8C,D). Similar to intact mice, ISO treatment caused an increase in LV mass in all groups of mice except the DOX-exposed gonadectomized males (Supplementary Figure S3).

## 3. Discussion

Cardiotoxicity still remains the most prominent limitation in DOX therapy for the treatment of cancers [1]. The use of low, repeated doses of DOX has reduced the risk of developing significant declines in heart function but is still associated with cardiac atrophy and the increased expression of markers for cardiac toxicity [7,21]. Due to these changes, the heart may become more susceptible to deterioration in cardiac function when exposed to a second cardiovascular stress. We used ISO as a means to induce cardiac stress without increasing the blood pressure. Sex has been documented as a contributing factor to the increased probability of developing severe cardiac toxicity, with males at greater risk than pre-menopausal females [15]. There have been no studies that show the sex difference in heart physiology in DOX-exposed mice that were subjected to a second cardiovascular stress later in life. We designed a treatment protocol where we administered low, repeated doses of DOX once a week for five weeks followed by a five-week recovery period before starting the 14-day ISO challenge. With this treatment protocol, we were able to identify sex differences after a 14-day ISO challenge in mice with prior DOX exposure.

Using the described treatment protocol, we did not observe any significant changes in cardiac function one or five weeks after the last dose of DOX, consistent with previous reports. In the report by Desai and colleagues (2021), they show that cumulative DOX doses below 24 mg/kg in B6C3F mice do not produce significant changes in heart function but are associated with reduced heart weight and elevated plasma troponin levels [5]. In the current project, we administered a total cumulative dose of 20 mg/kg, and no significant changes in cardiac function were observed. However, the heart size in males one week post-DOX treatment was significantly smaller compared to saline-treated mice. In contrast, female heart size appears to be similar between DOX and control mice, suggesting that the female sex protects from cardiac atrophy that is induced by DOX cumulative dose of 20 mg/kg.

The dose dependency in DOX-mediated cardiotoxicity is known by physicians, and in order to prevent DOX-mediated toxicity, low doses are administered to patients [1]. The use of low, repeated doses of DOX has contributed to reduced overt cardiac dysfunction; however, these patients have smaller and weaker hearts that predispose them to cardiovascular pathology later on in their life [22,23]. Hypertension is one of the major risk factors for developing overt cardiotoxicity in pediatric cancer survivors [6]. Identifying the mechanisms in which cardiovascular insults exacerbate DOX-mediated cardiotoxicity is important for the treatment and management of cardiovascular disease in cancer survivors. In our previous reports, we showed that angiotensin II, a pro-hypertensive ligand, was associated with significant deterioration in heart mass and function in DOX-exposed mice [7,9]. To explore the mechanisms of cardiac pathology without changes in blood pressure, we used ISO, which is a non-specific β-adrenergic receptor agonist known to induce inotropy and chronotropy in the myocardium and prolonged treatment is associated with deterioration in cardiac performance. Of note, β2-receptors in the vasculature cause vasodilation, ultimately reducing the resistance the heart has to work against [24,25]. We first assessed the acute effect of ISO on heart function in DOX-exposed mice (stress echocardiography). Five minutes after the first ISO treatment, there were significant increases in inotropy and chronotropy in control mice. In DOX-exposed mice, the inotropic response was maintained but the chronotropic response was blunted after acute ISO treatment. There were no apparent sex differences in the response to ISO between control and DOX-treated mice.

Interventions that increase sympathetic drive have been used to uncover latent cardiotoxicity in asymptomatic cancer patients who completed their DOX treatment. This has led to the use of stress echocardiography as a tool to assess cardiotoxicity in cancer survivors years after the cessation of DOX. Dobutamine [10,11,12,13] and exercise [14] both have been reported to be used in the clinic to perform stress echocardiography. Dobutamine is a β1-adrenergic receptor agonist that displays similar pharmacological actions to ISO. Exercise is known to increase inotropy and chronotropy similar to ISO. The use of stress echocardiography revealed reductions in LV wall thickness, which was associated with declines in cardiac function [10,11,12,13,14]. In the current report, we show that the inotropic response to ISO was maintained, but the chronotropic response was blunted in DOX-exposed male and female mice. We concluded that the acute ISO challenge did not reveal overt cardiomyopathy using a cumulative DOX dose of 20 mg/kg.

We then assessed the chronic effects of a 14-day ISO challenge in the control and DOX groups. Interestingly, significant reductions in cardiac function were observed in DOX-exposed male mice, whereas female mice did not produce any significant changes in cardiac function. The major finding of this study was that DOX-induced cardiac atrophy persisted even after the 14-day ISO challenge in male mice. Previous reports show that DOX administration resulted in smaller hearts in preclinical models. One report showed that juvenile mice exposed to DOX had a reduced heart weight in both male and female mice [26]. In experiments with adult rodents, males were more susceptible to experiencing significant reductions in heart weights compared to females [27,28]. In our report, we show that DOX-exposed male mice had smaller hearts five weeks after stopping DOX treatment, while female mice did not exhibit significant differences in heart size. The differences in the DOX effect on heart weight between reports may be due to differences in the age of mice when DOX was administered. The DOX-mediated reduction in heart weight can predispose male mice to cardiac deterioration when exposed to a second cardiac stressor. With regards to ISO, our laboratory reported that 14 days of ISO treatment at a dose of 10 mg/kg/day did not reveal any sex difference in cardiac function [29]. Therefore, we hypothesize that DOX pre-treatment can unmask sex differences in the response to ISO after a 14-day challenge. We have shown that ISO and angiotensin II failed to increase heart size in male mice exposed to a cumulative DOX dose of 12 mg/kg [7,9]. There is a paucity of information with regard to potential sex differences in the heart’s ability to respond to hypertrophic stimuli in DOX-exposed animals. Our current report is the first to compare the effects of ISO in the hearts of DOX-exposed male and female mice. We show, for the first time, that DOX-exposed male mice have smaller hearts compared to controls even after a 14-day ISO challenge. DOX-induced cardiac atrophy may be mediated by multiple mechanisms including p53-dependent inhibition of mammalian target of rapamycin (mTOR) [30] and forkhead box O1 (FOXO1)-mediated activation of muscle RING finger 1 (MuRF1) [31]. Additional pathways have been suggested to mediate DOX-induced cardiac atrophy including impaired insulin-like growth factor 1 (IGF-1) signaling, reactive oxygen species (ROS)-dependent upregulation of NADPH oxidase 2 (Nox2), altered PI3K-AKT pathway, and activated p38 MAPK (reviewed in [32,33]). Female mice were protected from DOX-induced cardiac atrophy and demonstrated a robust hypertrophic response to the ISO challenge. Similar to the observed sexual dimorphism in DOX-induced cardiac atrophy, female mice were protected from tumor-induced cardiac atrophy, which was attributed to enhanced inflammation and higher cardiac autophagy in male mice [34]. Generally, sexual dimorphism has been reported in pathologies causing muscle atrophy, with males more sensitive to inflammation-induced atrophic changes than females [35].

There were no significant sex differences in the gene expression for markers of cardiac hypertrophy, fibrosis, and inflammation. We did not observe sex differences in the gene expression of these markers in response to DOX alone or ISO in DOX-exposed mice, suggesting that sexual dimorphism in the hypertrophic response to ISO in DOX-exposed mice is not associated with sexually dimorphic expression of markers of cardiac hypertrophy, fibrosis, and inflammation. The prevention of an ISO-mediated hypertrophic response, accompanied by increased markers of cardiac toxicity, in male mice suggests that pre-exposure to DOX prevents the physiological adaptation to respond to pro-hypertrophic stimuli rather than preventing maladaptive cardiac remodeling. Counterintuitively, exposure to DOX at a young age (4 mg/kg/week for 5 weeks in this study) abrogated ISO-induced myocardial fibrosis in adult male and female mice. Although surprising, this finding is consistent with our earlier report that DOX (4 mg/kg/week for 3 weeks) partially abrogated ISO-induced cardiac fibrosis in adult male mice [9]. This apparent protective effect of DOX against ISO-induced cardiac fibrosis may be attributed to DOX-induced senescence and premature aging [36]. DOX causes cardiac fibroblast senescence with a decreased proliferation and migration [37,38]. In support of this notion, ISO treatment did not significantly exacerbate cardiac fibrosis in aged mice [39]. Similarly, aged mice show markedly reduced collagen deposition in response to myocardial infarction [40]. Nevertheless, the effect of DOX exposure on long-term exposure to ISO is yet to be determined in future experiments.

Another finding of this study was that deprivation of sex hormones, via gonadectomy, did not reverse the sexually dimorphic response to DOX. DOX-exposed castrated male mice exhibited a blunted hypertrophic response to ISO while ovariectomized female mice were able to maintain this response. Both gonadectomized male and female mice experienced declines in cardiac performance in response to ISO, with males having more significant declines. This finding is contrary to what we were expecting due to several lines of evidence suggesting the role of sex hormones in mediating sexual dimorphism in DOX-induced cardiotoxicity. Although several studies report the protective effects of estrogen against DOX-induced cardiotoxicity [17,41,42], there are also a few reports showing that androgen deficiency exacerbates DOX-induced cardiotoxicity [43,44]. This sheds light on the complexity of the sex-mediated effects of DOX on cardiotoxicity. Similar to this finding, gonadectomy did not abrogate sex differences in the fibrotic response to chronic ISO administration [45]. It is increasingly recognized that there are sex differences beyond endogenous hormone levels that can contribute to the observed sexual dimorphism. Several studies suggest that sex differences may be mediated genetically through the X and Y chromosomes [46,47,48] and/or prenatal hormone exposure [49]. Intriguingly, a recent study shows marked sex chromosome-dependent differences in cardiac proteomics that arise even before gonad formation [50]. Future studies are warranted to determine the relevance of these factors in mediating the sex differences observed in this model of DOX-induced cardiotoxicity.

## 4. Materials and Methods

### 4.1. Animals

All experimental procedures involving animals were approved by the University of Minnesota Institutional Animal Care and Use Committee (IACUC protocol number: 1807-36187A approved on 9 December 2018). We previously reported a lack of sex differences in ISO-induced cardiac dysfunction using control mice (21). Saline and ISO-treated DOX mice experiments ran in parallel to those groups of control mice. Intact and gonadectomized (GDX) male and female C57BL/6N mice were purchased from Charles River Laboratories at 4 weeks of age. Mice were given food and water ad libitum in a 14 h light/10 h dark cycle at 21 ± 2 °C. The mice were allowed to acclimate to the animal facility one week before starting experimental procedures. An illustration of the general experimental protocol is shown in Figure 1. Starting at 5 weeks of age, mice were administered intraperitoneal injections of DOX (4 mg/kg/week) or an equivalent volume of sterile saline for 5 consecutive weeks to reach a total cumulative dose of 20 mg/kg. Mice were not administered any pharmacological agent for a 5-week recovery period before starting the ISO treatment regime. ISO (10 mg/kg/day) or an equal volume of saline was administered to the mice using subcutaneous injections for 14 consecutive days. After the last day of the ISO injections, mice were humanely euthanized with decapitation under isoflurane anesthesia. Hearts were harvested, washed in ice-cold phosphate-buffered saline solution, flash-frozen in liquid nitrogen, and stored at −80°C for further analysis.

### 4.2. Echocardiography

Cardiac function was assessed with echocardiography using the Vevo 2100 system (VisualSonics, Inc., Toronto, ON, Canada) equipped with an MS400 transducer. Measurements of heart function were assessed 1 and 5 weeks after the last dose of DOX, immediately after the first dose of ISO (stress echo), and 1 day after the last ISO dose. Anesthesia was maintained using 2% isoflurane for the duration of the procedure. Mice were positioned in the supine position on a heated physiological monitoring stage. Parasternal short-axis images of the left ventricle were obtained in M-Mode at the level of the papillary muscles. Endocardial and epicardial borders were manually traced over 3–4 cardiac cycles and cardiac output (CO), heart rate (HR), stroke volume (SV), fractional shortening (FS), ejection fraction (EF), and left-ventricular (LV) mass were all determined using the Vevo 2100 cardiac measurement package.

### 4.3. Histopathology

Left ventricular heart sections were collected, fixed in 10% neutral buffered formalin, and embedded in paraffin. Four-micron sections were stained with Masson’s trichrome stain to assess myocardial fibrosis. The severity of fibrosis on the trichrome-stained section was assessed as follows: 0, absent; 1, minimal fibrosis; 2, mild fibrosis; 3, moderate fibrosis; and 4, marked fibrosis. The assessment was performed by two persons who were blinded to the experimental groups. The average of the two independent assessments was reported.

### 4.4. RNA Extraction and Real-Time PCR

Total RNA was extracted from frozen hearts using TRIzol^®^ reagent (Life Technologies, Carlsbad, CA, USA), according to the manufacturer’s instructions. RNA concentrations were quantified at 260 nm using a NanoDrop 8000 spectrophotometer (Thermo Fisher Scientific, Wilmington, DE, USA). First-strand cDNA was synthesized from 1.5 μg total RNA using the high-capacity cDNA reverse transcription kit (Applied Biosystems, Foster City, CA, USA) according to the manufacturer’s instructions. Real-time polymerase chain reaction (PCR) was used to measure specific mRNA expression with PCR amplification of the synthesized cDNA using 384-well optical reaction plates in an ABI 7900HT instrument (Applied Biosystems, Foster City, CA, USA). The 20 µL reaction mix contained 1 µL of cDNA sample, 0.025 µL of 30 µM forward primer and 0.025 µL of 30 µM reverse primer (40 nM final concentration of each primer), 10 µL of SYBR Green Universal Mastermix (Life Technologies, Carlsbad, CA, USA), and 8.95 µL of nuclease-free water. Thermocycler conditions were as follows: 95 °C for 10 min, followed by 40 PCR cycles of denaturation at 95 °C for 15 sec, and annealing/extension at 60 °C for 1 min. Gene expression was determined using primers from previously published studies that are listed in Table 2. The mRNA expression levels were normalized to beta-actin, and the relative expressions were calculated using the ΔΔCt method.

### 4.5. Statistical Analysis

All data are presented as mean ± SEM. Data were analyzed using a two-way analysis of variance (ANOVA) with a Šidák post hoc test. A *p*-value < 0.05 was noted to be statistically significant. *p*-values < 0.1 were reported as numerical values.

## 5. Conclusions

We show, for the first time, the effect of chronic ISO administration in DOX-exposed male and female mice. Doxorubicin-exposed male mice are more susceptible to cardiac atrophy even after a 14-day ISO challenge when compared to control mice. Female mice do not exhibit cardiac atrophy after DOX administration and experience a robust hypertrophic response to ISO that is similar to that observed in control mice. The sex-specific effect of ISO on the hypertrophic response is associated with greater deterioration in cardiac performance, presumably due to increased sheer stress on the smaller heart. Pre-exposure to DOX abrogated ISO-induced cardiac fibrosis in both male and female mice. However, markers of pathologic hypertrophy were elevated in males and females, suggesting that the prevention of hypertrophy in male mice is a maladaptive process. Furthermore, the observed sex difference was not reversed in mice without sex hormone-producing organs. Therefore, we conclude that the mechanisms that produce this sex difference are largely independent of sex hormones.

## Figures and Tables

**Table 1:** **Baseline heart function one and five weeks after the last DOX injection in intact mice.** Heart function was assessed with echocardiography in intact males (n = 6–7) and females (n = 7). Values are represented as means ± SEM. Statistical significance of pairwise comparisons was determined using two-way ANOVA with Šidák’s post hoc analysis. Statistical significance was noted when *p* < 0.05 for comparisons between saline and DOX (*). Abbreviations: DOX, doxorubicin; CO, cardiac output; HR, heart rate; SV, stroke volume; FS, fractional shortening; EF, ejection fraction; LVESV, left ventricular end systolic volume; LVEDV, left ventricular end diastolic volume; LV, left ventricular; LVAW;s, left ventricular anterior wall systolic; LVAW;d, left ventricular anterior wall diastolic; LVPW;s, left ventricular posterior wall systolic; LVPW;d left ventricular posterior wall diastolic.

Parameter	1-Week Post DOX				5-Week Post DOX			
	Male		Female		Male		Female	
	Saline	DOX	Saline	DOX	Saline	DOX	Saline	DOX
CO (mL/min)	15.56 ± 0.55	13.07 ± 0.64	13.21 ± 1.06	13.17 ± 0.89	15.31 ± 0.78	14.16 ± 0.52	11.65 ± 0.96	13.26 ± 0.65
HR (BPM)	363 ± 8	340 ± 14	366 ± 14	362 ± 13	378 ± 15	377 ± 15	376 ± 15	372 ± 17
SV (µL)	42.85 ± 1.41	38.48 ± 0.79	35.75 ± 1.84	36.13 ± 1.25	40.65 ± 1.84	37.72 ± 1.16	30.89 ± 2.13	35.68 ± 0.84
FS (%)	30.10 ± 1.82	31.73 ± 2.36	29.86 ± 1.56	32.15 ± 32.15	32.64 ± 1.64	33.59 ± 2.46	29.09 ± 3.30	34.81 ± 1.13
EF (%)	57.48 ± 2.82	59.90 ± 3.66	57.39 ± 2.36	60.93 ± 1.63	61.42 ± 2.41	62.59 ± 3.63	55.75 ± 4.99	64.80 ± 1.57
LVESV (µL)	31.46 ± 4.22	26.59 ± 3.93	27.34 ± 2.63	23.75 ± 1.67	25.48 ± 4.17	24.65 ± 4.75	26.09 ± 4.58	19.43 ± 1.28
LVEDV (µL)	73.00 ± 3.72	63.44 ± 3.43	61.17 ± 3.00	59.37 ± 2.69	64.50 ± 5.55	60.87 ± 6.91	53.58 ± 5.06	54.45 ± 1.43
LV Mass (mg)	80.06 ± 4.03	66.85 ± 3.63 *	66.69 ± 3.84	65.96 ± 2.81	82.02 ± 1.97	69.75 ± 5.01	63.49 ± 4.82	63.17 ± 2.97
LVAW;s (mm)	1.11 ± 0.092	1.04 ± 0.075	0.94 ± 0.061	0.90 ± 0.055	1.33 ± 0.076	1.11 ± 0.29 *	0.92 ± 0.073	1.00 ± 0.044
LVAW;d (mm)	0.75 ± 0.037	0.71 ± 0.060	0.595 ± 0.040	0.580 ± 0.043	0.85 ± 0.036	0.70 ± 0.025 *	0.68 ± 0.046	0.63 ± 0.037
LVPW;s (mm)	1.01 ± 0.057	0.96 ± 0.051	0.93 ± 0.057	0.99 ± 0.034	1.09 ± 0.046	1.00 ± 0.019	0.90 ± 0.053	1.02 ± 0.045
LVPW;d (mm)	0.64 ± 0.017	0.59 ± 0.032	0.69 ± 0.041	0.67 ± 0.021	0.66 ± 0.023	0.59 ± 0.048	0.57 ± 0.023	0.64 ± 0.026

**Table 2:** Primer sequences used for real-time PCR experiments.

Gene ID	Forward Primer	Reverse Primer
*Anp*	GGAGCCTACGAAGATCCAGC	TCCAATCCTGTCAATCCTACCC
*Col1a1*	CTGGCGGTTCAGGTCCAAT	TTCCAGGCAATCCACGAGC
*Col3a1*	ATGGTGGTTTTCAGTTCAGCTATG	GCCCGGCTGGAAAGAAGT
*Galectin-3*	TATCCTGCTGCTGGCCCTTAT	GTTTGCGTTGGGTTTCACTG
*Cox2*	CTGGTGCCTGGTCTGATGATG	GGCAATGCGGTTCTGATACTG
*Tnf-ɑ*	CCAGACCCTCACACTCAGATCA	CACTTGGTGGTTTGCTACGAC
*β-actin*	TATTGGCAACGAGCGGTTCC	GGCATAGAGGTCTTTACGGATGTC

## Data Availability

Data is contained within the article and supplementary material.

## Supplementary Materials

The following supporting information can be downloaded at: www.mdpi.com/xxx/s1-3, Figure S1: Magnitude of change following stress echocardiography; Figure S2: Magnitude of change in echocardiography parameters following 14-day ISO treatment in DOX-exposed intact mice.; Figure S3: Magnitude of change in echocardiography parameters following 14-day ISO treatment in DOX-exposed gonadectomized mice.
