# Supplementary material for: Exposure to Doxorubicin Modulates the Cardiac Response to Isoproterenol in Male and Female Mice
Source: Pharmaceuticals (Basel). 2023 Mar 4;16(3):391. doi: 10.3390/ph16030391 (PMC10058259; doi:10.3390/ph16030391)
Supplement: Supplementary file 1 [file pharmaceuticals-16-00391-s001.zip › pharmaceuticals-2133219-supplementary.pdf]

Supplementary Figure S1

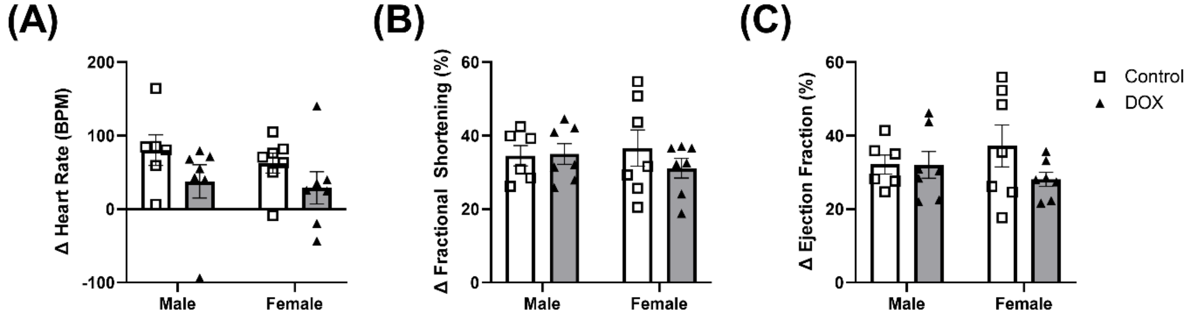

Figure S1. Magnitude of change following stress echocardiography. Echocardiography was performed before and five minutes after the first ISO injection. Change in A) heart rate, B) fractional shortening, and C) ejection fraction were determined by subtracting values obtained following ISO treatment from baseline in intact males (n=6-7) and females (n=7) respectively. Values are represented as means ± SEM. Statistical significance of pairwise comparisons was determined by two-way ANOVA with Sidak's post-hoc analysis. No significant differences were observed. Abbreviations: DOX, doxorubicin; ISO, Isoproterenol.

Supplementary Figure S2

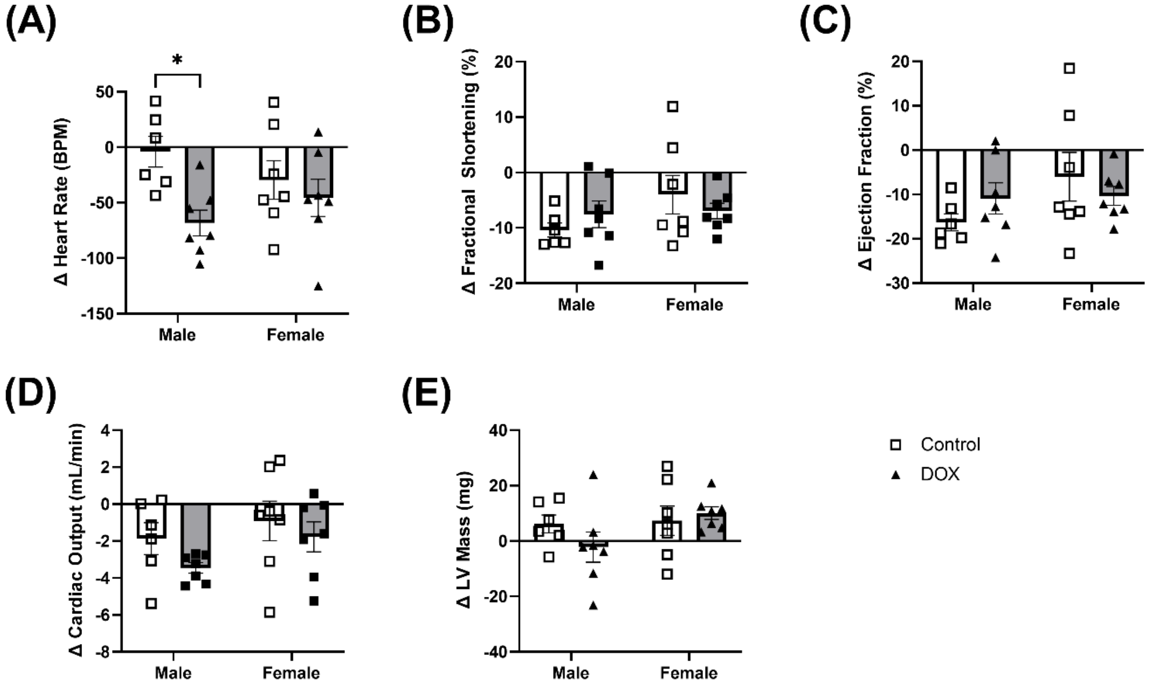

Figure S2. Magnitude of change in echocardiography parameters following 14-day ISO treatment in DOX-exposed intact mice. Echocardiography was performed before and after the 14-day ISO challenge. Change in A) heart rate, B) fractional shortening, C) ejection fraction, D) cardiac output, and E) LV Mass were determined by subtracting values obtained following ISO treatment from baseline in intact males (n=6-7) and females (n=7). Values are represented as means ± SEM. Statistical significance of pairwise comparisons was determined by two-way ANOVA with Sidak's post-hoc analysis. Statistical significance was noted when $p<0.05$ for comparisons between DOX and control (*). Abbreviations: DOX, doxorubicin; ISO, Isoproterenol; LV, left ventricle.

Supplementary Figure S3

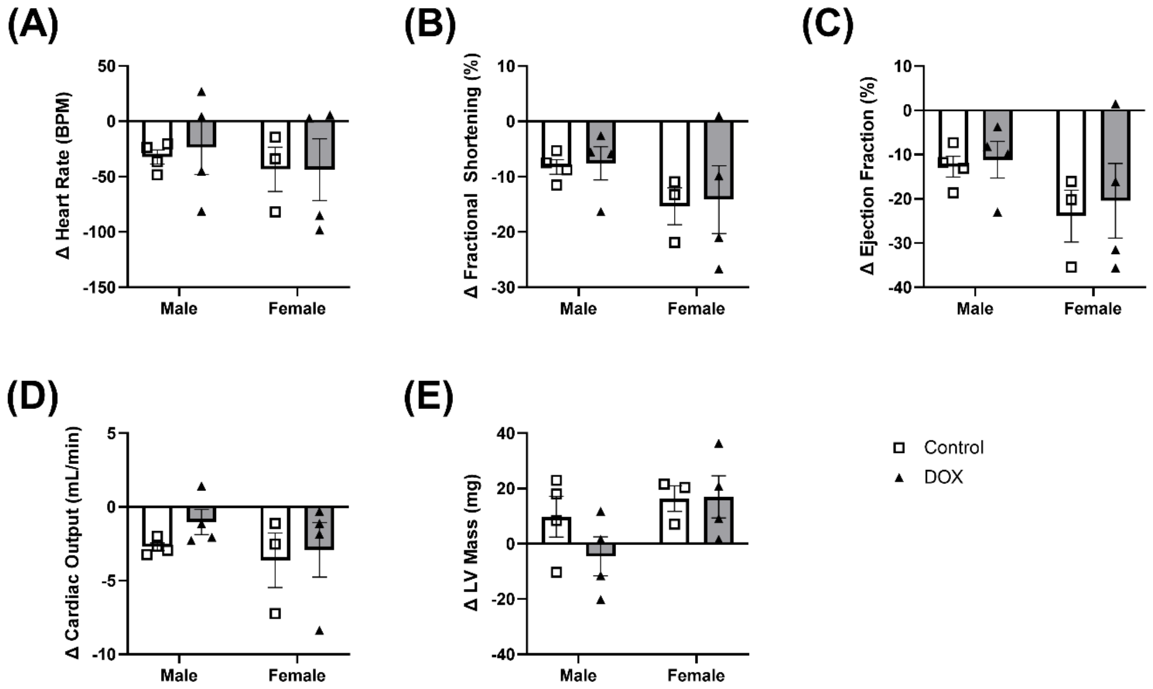

Figure S3. Magnitude of change in echocardiography parameters following 14-day ISO treatment in DOX-exposed gonadectomized mice. Echocardiography was performed before and after the 14-day ISO challenge. Change in A) heart rate, B) fractional shortening, C) ejection fraction, D) cardiac output, and E) LV Mass were determined by subtracting values obtained following ISO treatment from baseline in gonadectomized males (n=4) and females (n=3-4). Values are represented as means ± SEM. Statistical significance of pairwise comparisons was determined by two-way ANOVA with Sidak's post-hoc analysis. No significant differences were observed. Abbreviations: DOX, doxorubicin; ISO, Isoproterenol.
